# Onco-TESE (Testicular Sperm Extraction): Insights from a Tertiary Center and Comprehensive Literature Analysis

**DOI:** 10.3390/medicina59071226

**Published:** 2023-06-29

**Authors:** Lorenzo Cirigliano, Marco Falcone, Murat Gül, Mirko Preto, Carlo Ceruti, Natalia Plamadeala, Federica Peretti, Ilaria Ferro, Martina Scavone, Paolo Gontero

**Affiliations:** 1 Urology Clinic—A.O.U. “Città della Salute e della Scienza”—Molinette Hospital, University of Turin, 10100 Turin, Italy; dr.lorenzocirigliano@gmail.com (L.C.);; 2 Neurourology Clinic—A.O.U. “Città della Salute e della Scienza”—Unità Spinale Unipolare, 10100 Turin, Italy; 3 Department of Urology, School of Medicine, Selcuk University, Konya 42005, Turkey

**Keywords:** onco-TESE, male infertility, testicular cancer, cryopreservation, sperm, TESE

## Abstract

*Background and Objectives*: The peak of incidence of testicular cancer (TC) occurs among individuals in their reproductive age, emphasizing the importance of fertility preservation as an integral aspect of disease management. Sperm cryopreservation performed before orchiectomy is ineffective in azoospermic men, necessitating alternative approaches such as microdissection testicular sperm extraction (mTESE) at the time of orchiectomy (onco-mTESE) to obtain viable sperm. This study presents the findings from our institution’s experience with onco-mTESE and critically discusses our results in light of the existing body of literature. *Materials and Methods*: This is a tertiary center retrospective analysis of onco-mTESE procedures performed at a single center between December 2011 and July 2022. The included patients were post-puberal men with testicular tumors requiring orchiectomy, along with concomitant severe oligozoospermia or azoospermia. Bilateral mTESE was performed in all cases. Surgical outcomes, sperm retrieval rates, the usage of preserved viable sperm, assistive reproductive techniques’ results, and post-operative serum testosterone were recorded. *Results*: A total of nine patients were included, with a median age of 34 (IQR 29–36) years. All patients had germ cell tumors (GCTs), with seminomatous and non-seminomatous GCTs accounting for 44.4% (*n* = 4) and 55.6% (*n* = 5) of patients, respectively. Sperm retrieval occurred in three (33%) patients: one patient in the ipsilateral testis, one in the contralateral testis, and one in both testes. No complications were reported during the procedure, and no post-operative hypogonadism was observed. Among the three patients with successful sperm retrieval, an intracytoplasmic sperm injection (ICSI) was performed in two patients, resulting in two pregnancies, leading to one healthy live birth and one miscarriage. *Conclusions*: In the context of TC, it is essential to conduct a thorough evaluation of testicular function, including a semen analysis and cryopreservation. Onco-mTESE has proven its safety in preserving fertility in azoospermic cases while ensuring the efficacy of oncological treatment.

## 1. Introduction

Testicular cancers (TCs) represent 1% of all malignancies in men [1], with a peak incidence in the fourth decade for seminomatous germ cell tumors (SGCTs) and in the third decade for non-seminomatous germ cell tumors (NSGCTs) [2]. Over the past few decades, the incidence of TC has gradually increased, particularly in industrialized countries [3]. Despite this increase, advances in diagnostic accuracy and improved therapeutic interventions have led to a substantial improvement in the survival rates of individuals affected by this disease. These advancements have led to earlier detection, a more precise diagnosis, and the implementation of effective treatment strategies, ultimately resulting in improved outcomes for patients with TC. Currently, the 5-year cancer-specific survival (CSS) for SGCTs and NSGCTs is high, reaching 95% and 96%, respectively, in the good-prognosis group [4]. However, in the poor-prognosis group, excluding SGCT cases, the 5-year CSS declines to 67% [5].

Given the young age and the high rates of CSS, the preservation of fertility is a significant aspect of TC treatment that can potentially lead to a temporary or permanent decline in fertility [6]. This issue becomes even more significant considering the increasing prevalence of infertility among couples, especially in industrialized nations, as well as the fact that testicular cancer itself is a risk factor for infertility [7]. A considerable amount of patients (around 50%) diagnosed with TC present abnormalities in seminal fluid parameters, even before initiating treatment [8]. These alterations can vary in severity, ranging from mild to severe, with azoospermia observed in approximately 10–15% of cases [9,10]. International guidelines, therefore, strongly advocate for the thorough and detailed counselling of the potential complications, including the irreversible possibilities, caused by TC treatments.

Sperm cryopreservation is widely recognized as an effective method of preserving male fertility. Prior to undergoing orchiectomy, a semen sample can be obtained through masturbation and subsequently cryopreserved for future use. In cases of azoospermia, cryptozoospermia, or failure to retrieve sperm (due to medical, cultural, or religious factors), an alternative method called testicular sperm extraction (TESE) can be employed [11]. This technique can be performed with the aid of an operating microscope (mTESE) in order to select the most promising seminiferous tubules, enabling the retrieval of viable sperm. In cases of azoospermia accompanied by TC, onco-TESE, a sperm extraction procedure either with a microscope or using conventional methods, can be used to preserve fertility; however, data regarding onco-TESE are currently limited in the literature.

The primary objective of our study is to present the outcomes of the onco-TESE results from our center. By combining our center’s experiences with the insights gleaned from recent studies, we seek to provide a comprehensive analysis of the current landscape of fertility preservation in the context of testicular cancer.

## 2. Materials and Methods

### 2.1. Study Setting and Patients

A retrospective analysis was conducted on patients diagnosed with TC who underwent onco-mTESE at a single tertiary referral center from December 2011 to July 2022. The data for this study were retrospectively extrapolated from clinical records.

This study included post-puberal adults with TC who required orchiectomy and had concomitant azoospermia or severe oligozoospermia (defined as <1 million sperm/mL) or were unable to provide a semen sample. Patients with incomplete clinical records or follow-up were excluded from this study. Pre-operative centrifuged semen pellet analyses were performed to confirm azoospermia, and all patients underwent a testicular ultrasound (US) before surgery.

The onco-mTESE procedures were performed ex vivo on both testicles if the patients were not monorchid, and they were performed by the same surgeon in the operating room under general or spinal anesthesia. An inguinal approach was utilized for the testicle with the tumor, while a median scrotal approach was fashioned to reach the healthy contralateral testicle. The tunica albuginea was transversely opened approximately 1.5 cm away from the tumor or at the center on the healthy testis. Tissue samples were then collected under magnification (20–25×) using an operative microscope and sent to the laboratory of pathology and reproductive biology for further analysis. 

Surgical outcomes, including operative time, hospital stay, sperm retrieval rates (SRRs), and post-operative complications (defined according to the Clavien–Dindo classification [12]), were recorded. The patients were questioned during outpatient visits or via telephone calls regarding the use of cryopreserved sperm vials. This study adhered to the principles outlined in the Declaration of Helsinki, and written informed consent for the procedure and publication of medical data was obtained from all patients. Ethical approval was obtained from the Institutional Review Board of Molinette Hospital, University of Turin (Protocol Number 51.705).

### 2.2. Outcome Measures and Statistics

The normality of variable distributions was tested using the Kolmogorov–Smirnov test. Categorical variables are described using frequencies and percentages. Differences between groups were assessed using the Chi-square test or Fisher’s exact test, depending on the expected cell counts. For continuous variables with a normal distribution, the mean and standard deviation (SD) are reported, whereas variables with a non-normal distribution are described using the median and interquartile range (IQR). Differences between groups were assessed using Student’s *t*-test for normally distributed variables and the Mann–Whitney U test for non-normally distributed variables. A *p*-value <0.05 was considered statistically significant. 

Recurrence-free survival (RFR) was estimated using a Kaplan–Meier analysis. The log-rank test was employed to identify any significant differences in terms of survival between groups. Statistical analyses were performed using the Statistical Package for the Social Sciences (SPSS; v. 28; IBM, Chicago, IL, USA).

## 3. Results

A total of 422 patients underwent orchiectomy from December 2011 to July 2022. Among them, 13 were found to be azoospermic on a semen analysis, but only 9 were interested in preserving fertility. Those 9 patients met the inclusion criteria and were included in our study and underwent onco-mTESE. The median age of the patients was 34 years (IQR: 29–36). Detailed demographic and pathological characteristics are presented in Table 1 and Table 2, respectively.

All nine lesions were hypoechogenic and vascularized on color Doppler US. Azoospermia was observed in all patients, and onco-mTESE was performed bilaterally in six subjects (66.7%), while three patients (33.3%) had a solitary testis. Among the patients, three (33.3%) had a previous history of TC. Testis and tumor characteristics are described in Table 3.

The lesions were located in the left testis in eight cases (88.9%) and in the right testis in one subject (11.1%); the median testis size was 8 mL (IQR: 7.5–13), while the median lesion size was 5 mL (IQR: 4–5.5). All nine cases (100%) were histologically confirmed as having GCTs. The majority of GCTs identified were NSGCTs (56.6%), including one case (11.1%) of a teratoma, one case (11.1%) of a yolk sac tumor, and three cases (33.3%) of a mixed GCT.

Spermatozoa were successfully retrieved in three out of the nine cases (33.3%). Among these, in one patient (11.1%), positive sperm retrieval occurred bilaterally; another patient (11.1%) had sperm retrieval only in the non-tumoral testis; and one patient (11.1%) had sperm extraction only in the tumoral testis, while no sperm were found in the contralateral testis. The median Johnsen score was 2 (IQR = 1.5–7). The cryopreserved spermatozoa retrieved from the patients were used for an intracytoplasmic sperm injection (ICSI) in two cases, resulting in one miscarriage in the 6th month of pregnancy and one healthy live birth. 

No bilateral tumor cases were reported. No significant difference was observed in SRRs between patients with SGCTs and NSGCTs (*p* = 0.86). No intraoperative complications were observed, and no significant post-operative complications (>Clavien-Dindo III) were reported. Post-orchiectomy testosterone levels remained within normal values in patients who still had a testis, with a pre-operative level of 18 nmol/L and a post-operative level of 15.5 nmol/L. 

The average duration of the operation was 78 min (IQR = 70–84), and the average length of stay was 1 day (IQR: 1-1). At the last follow-up, one patient (11.1%) died due to TC, while all eight living patients (88.9%) followed for cancer were in oncological remission. Importantly, performing onco-mTESE did not lead to any delays in the administration of oncological treatment for any of the patients.

## 4. Discussion

TC can occur in young males, typically presenting as a palpable mass on self-examination or during a testicular ultrasound study [13]. The treatment options for testicular tumors have improved over the years, resulting in better overall survival and cancer-specific survival of patients. However, many young patients who survive TC may face fertility issues, as the rates of oligozoospermia and azoospermia are high in this population [11,14]. Therefore, it is essential to inform patients about the potential impacts of TCs and their treatments on fertility, as some may have an interest in seeking offspring in their lifetime [10,15,16]. In this study, we demonstrated that onco-TESE is safe, as no adverse events or delay in oncological treatment occurred, and we proved that it was an effective option for preserving fertility in 33.3% of the azoospermic patients with TC.

## 4.1. TC and Fertility 

In patients with TC, several factors have been associated with infertility. Testicular dysgenesis syndrome (TDS), first described in 2001, is a condition that may contribute to the development of fertility issues in patients with TC [17]. According to the TDS hypothesis, testicular cancer is one of the symptoms, in addition to other phenotypes, including cryptorchidism, hypospadias, a shortened anogenital distance, reduced Leydig cell function, and decreased spermatogenesis due to disrupted embryonic gonadal development. TDS is supported by histological studies observing that men referred to andrology clinics for fertility issues frequently exhibit dysgenetic characteristics, such as undifferentiated tubules with visibly immature Sertoli cells, clusters of poorly differentiated tubules, or hyaline bodies, which are frequently associated with testicular cancer. Although not all cases of genital deformities and infertility are a part of TDS, milder phenotypes linked to altered testis development in fetal life due to various environmental or lifestyle-related causes might be considered part of TDS. The clinical expression of TDS may vary considerably; a thorough estimate of the frequency of medium-severity TDS in the male population is 5% [17,18,19,20]. 

Moreover, TC itself may interfere with normal spermatogenesis, and the closer the tumor to the testis, the more likely it is to affect spermatogenesis [21]. This phenomenon has only been observed in malignant tumors, while it does not occur in benign testicular lesions [22]. The inflammation and oxidative stress caused by the tumor can also disrupt seminiferous tubules adjacent to the tumor [23]. Tumor size has been correlated with residual spermatogenesis, with tumors larger than 4 cm being associated with worse rates of spermatogenic defects [21]. Moody et al. [11] observed that, in men with testis tumor occupation >50%, there was a significant decrease in the likelihood of spermatogenesis in the affected testis. In their series of 103 patients with GCTs, spermatogenesis was observed away from the tumor in 50% of the patients, whilst spermatogenesis close to the tumor and within it was observed in 4% and 1% of cases, respectively. 

Testicular Microcalcification (TM) has been proposed as a common element in TC-related infertility, and its role has been extensively debated in the literature. No significant conclusion has been reached yet, and the relationship of finding US microlithiasis with infertility and TC remains unclear [24,25]. The current guidelines advise men with TM to undergo self-examination, as this may even result in the early diagnosis of a testicular GCT, while infertile males with TM who are in one of the higher risk groups may be offered a testicular biopsy [26]. 

This evidence, except for TDS, does not explain why defects in spermatogenesis have been observed in the unaffected, contralateral testis. A different systemic mechanism of tumor-induced subfertility has been proposed. Hormones released locally by the tumor, such as beta-human chorionic gonadotropin (b-HCG), alpha-fetoprotein (AFP), and lactate dehydrogenase (LDH), are hypothesized to increase estradiol levels in the testis, thus affecting spermatogenesis, as estrogens may inhibit the high intratesticular testosterone concentrations required for spermatogenesis [27,28]. However, increased tumor marker levels (b-HCG, AFP, and LDH) are not accurate indicators of spermatogenesis impairment [21]. Testicular tumors cause a breach in the blood–testis barrier. This barrier blocks autoantibodies from developing against sperm, and barrier lesions may thus induce the formation of antisperm antibodies, thus damaging spermatogenesis bilaterally [28]. The correct production of functional sperm relies on a perfect balance in the complex hypothalamic–pituitary axis. Testicular cancer may affect the hypothalamic–pituitary axis, most commonly through elevations in serum human chorionic gonadotropin and estradiol, causing a decrease in follicle-stimulating hormone (FSH) [11,29]. Finally, malnutrition and fever, which are common in patients with cancer, may be linked to spermatogenic changes, resulting in substantially reduced sperm concentrations or even azoospermia [30].

Therapies for TC itself can also cause fertility issues. Adjuvant chemotherapy after orchiectomy is often indicated. Spermatogenesis is compromised, as chemotherapy drugs cross the testis–blood barrier, and subsequent damage to dividing cells leads to a significant reduction in sperm concentration up to azoospermia. Cisplatin-based combination therapy is recommended by European guidelines [13]. The standard adjuvant treatment for GCTs in stage I is based on the combination of bleomycin, etoposide, and cisplatin (BEP) administered as one course, and a one-year full recovery of testicular function has been described [31]. A correlation between the cumulative chemotherapy dose and infertility has been established [32] For more prolonged therapy schemes used in more advanced diseases, the recovery of spermatogenesis within 4 years is possible [33], but the results in the literature are not univocal [34].

Radiation therapy is also considered a serious threat to fertility preservation, as testicular tissue is particularly sensitive to radiation, considering that less than 0.5 Gy can cause reversible oligozoospermia, while <3 Gy leads to reversible azoospermia. For higher doses of radiation, the resulting azoospermia can be irreversible, even though the radiation is not directly aimed at the testicles, as the damage is determined by the scattered radiation [6]. The current European guidelines suggest to adopt contraception for at least six months after the completion of both chemotherapy and radiotherapy [13], as the possible teratogenic effect deriving from those therapies [35] makes it highly inadvisable to seek conception in the time following adjuvant therapies. 

## 4.2. The Role of Onco-TESE in Patients with TC 

Clinicians should advise patients with TC to undergo a seminal fluid evaluation with the possibility of cryopreservation before undergoing any gonadotoxic treatment, including surgery [36]. Cryopreserved sperm can be used in ICSIs in cases where infertility develops after treatment, even though cryopreservation techniques may alter sperm quality [37,38]. Retaining fertility and having the option to have children are important aspects of life for TC survivors [39].

However, in clinical practice, patients often do not receive adequate counseling regarding the impact of different treatments on fertility and the option of sperm cryopreservation [40]. Only about one-third of patients with TC bank their sperm [41]. The reasons for not undergoing semen cryopreservation include a lack of interest, a desire to start chemotherapy as soon as possible, the completion of family planning, inadequate pre-operative counseling, and a lack of awareness about how treatment may affect future fertility [42]. Since there are currently no reliable predictive factors to determine azoospermia in patients with TC at diagnosis or after treatment, and cryopreservation before chemotherapy or radiation therapy is more cost-effective than deferred treatment [43], cryopreservation should always be encouraged in patients with TC before active treatment, unless it significantly delays cancer treatment [44]. As semen quality may improve after surgery, experts recommend offering cryopreservation both before and after orchiectomy to maximize the quality of semen samples [45]. Performing a pre-operative semen analysis is crucial to reduce the risk of post-operative azoospermia and to identify men with pre-existing azoospermia.

While a 48 h abstinence period is recommended for elective semen assessment, it is not necessary in the context of cryopreservation. A semen sample can be collected within an hour at the lab or through masturbation at the facility. If masturbation is not possible due to medical, cultural, or religious reasons, alternative methods, such as electroejaculation, vibratory-induced ejaculation, percutaneous aspiration of the seminal vesicles, or testicular biopsy, can be considered. In cases where pre-operative sperm cryopreservation is not feasible due to azoospermia, severe oligospermia, or difficulty in obtaining semen, performing a surgical collection of spermatozoa simultaneously with testicular surgery through onco-TESE may increase the chances of reproduction for young individuals affected by TC.

In patients with TC, sperm cryopreservation sometimes cannot be performed pre-operatively for azoospermia, severe oligospermia, or in the case of difficulty in obtaining semen. In this context, performing a surgical collection of the spermatozoa concomitantly with testicular surgery through onco-TESE may increase the chances of reproduction of young individuals affected by TC. 

The first reported case of onco-TESE, where a testicular biopsy and the cryopreservation of viable sperm were performed prior to orchiectomy for TC, was described by Res et al., in 1997 [46]. Subsequently, the term onco-TESE was introduced by Schrader et al. [47] in 2003, when they published the largest retrospective series on onco-TESE, including 31 azoospermic patients with TC. This series also included non-germ-cell tumors, such as Hodgkin’s disease (*n* = 7) and non-Hodgkin’s lesions (*n* = 10). 

During onco-TESE, a sperm analysis can be performed in the operating room by examining seminiferous tubules from non-tumor-bearing regions using a benchtop microscope. If viable sperm are found, they are analyzed and cryopreserved. In cases where no or inadequate sperm are detected, contralateral TESE can be performed. In our center, we perform a sperm analysis in a separate laboratory, allowing us to directly perform modified TESE on both the tumor-affected testis and the contralateral testis. A biopsy of the contralateral testis is useful for improving the retrieval of spermatozoa and can also help exclude the presence of a simultaneous carcinoma in situ.

A comprehensive review of the literature identified a total of 22 [46,47,48,49,50,51,52,53,54,55,56,57,58,59,60,61,62,63,64,65,66,67] articles evaluating onco-TESE (testicular sperm extraction) prior to the initiation of gonadotoxic treatment (Table 4). Among these articles, the majority (*n* = 15) were case reports or case series, while seven were retrospective investigations. In total, 130 onco-TESE procedures were performed, with 99 of them conducted on azoospermic individuals. The retrospective studies reported positive sperm retrieval (SR) rates ranging from 43% to 83% (38.1% [55], 45.2% [47], 58.3% [66], 66.7% [54], 78% [48], 80% [52], and 83% [65]), indicating the successful retrieval of spermatozoa. The case reports mainly focused on describing the TESE procedures that resulted in the retrieval and cryopreservation of spermatozoa. The utilization of retrieved sperm was evaluated in five retrospective studies and eight case reports, covering a total of 21 ICSI cycles, which led to 17 pregnancies or live births. Among the different techniques used, TESE was the most frequently performed procedure (95 cases), followed by mTESE with 36 cases.

Hallack et al. [52] conducted a retrospective analysis at a single center, involving five patients. Among these cases, four patients had benign Leydig cell tumors, including a patient with bilateral tumors, while one patient had a SGCT. The study did not specify whether sperm retrieval occurred only in cases of benign lesions, but it reported a positive sperm retrieval in four out of the five patients. No intraoperative complications were observed, and the mean testicular volume measured via an ultrasound after resection was not significantly different from the pre-operative values (*p* = 0.32).

In the study by Furuhashi et al. [54], six patients were included in a monocentric series. Among them, four patients were azoospermic, and two were oligozoospermic. Five patients were found to have a germ cell tumor (GCT) based on histological examination. However, the study did not provide data on post-operative complications. Among the four patients with positive SR, two patients underwent ICSI, resulting in pregnancies, although information on live births was not reported.

In a single-center retrospective analysis by Berookhim and Mulhall [55], sperm retrieval strategies for fertility preservation in males undergoing cancer treatment were evaluated. The study included a total of 49 patients, of whom only 8 had testicular germ cell tumors (TGCs) and 2 had benign Leydig cell tumors. The details regarding the number of patients with TC or those who required chemotherapy or radiotherapy for other malignancies were not provided. Among the included patients, 21 cases of TESE were described. However, it is unclear how many of these cases were specifically related to TC or other malignancies. Three patients underwent ex vivo TESE on the testis affected by TC immediately after orchiectomy, but none of them had sperm retrieved. Additionally, a total of 18 patients underwent both TESE and electroejaculation. The study did not report any complications associated with TESE procedures.

Blecher et al. [65] conducted a multicenter retrospective review in Australia, involving a total of 13 patients. Among these patients, seven had GCTs, four had non-germ-cell tumors (non-GCTs), and two had benign lesions, specifically Leydig cell hyperplasia and fibrous scars. The study reported no significant complications associated with the procedure, and the mean testosterone levels before and after orchiectomy were 12.0 nmol/L and 13.5 nmol/L, respectively. Sperm utilization involved both cryopreserved (four cases) and fresh (two cases) sperm. Six pregnancies were achieved, resulting in five healthy live births and one miscarriage.

Giwerc et al. [66] published a single-center retrospective study in 2021, involving 24 patients. The study reported on GCTs, and onco-TESE procedures were performed using different approaches based on the tumor location. For unilateral cancer, a contralateral scrotal approach was used on the healthy testicle, while an inguinal approach was used for a single testicle or bilateral cancer. The study found that the rate of semen collection failure was 70% (7 out of 10 patients) in patients, with reasons such as erectile dysfunction, the deterioration of general condition, anorgasmia, and cultural or religious refusal. In patients with severe oligozoospermia/cryptozoospermia, the rate was 80% (four out of five patients), while in those with azoospermia, it was 33.3% (three out of nine patients). Bilateral onco-TESE was performed in two subjects: one patient with synchronous bilateral TC and small testes, and another patient with metastatic disease and a “burned-out testis.” Both cases showed positive SR. The study reported two post-operative complications: one case of purulent melting of the biopsied testicle leading to emergency orchiectomy (Clavien-Dindo III) and one case of acute urinary retention (Clavien-Dindo I). Among the patients, one (7.14%) used the retrieved sperm for ICSI, and three patients had children using donor sperm from a sperm bank [65].

Scott et al. [48] conducted a multicenter retrospective study focusing on fertility preservation in patients with TC. Out of the 61 patients included in the study, 9 underwent onco-TESE due to azoospermia. Serum LDH was found to be a predictor of azoospermia ((OR 1.02 (1.00–1.02), *p* < 0.01) in a univariate analysis, and this was confirmed in a multivariate analysis (1.03 (95% CI 1.01–1.05, *p* < 0.01)). However, the paper did not provide specific information regarding the histology of the tumors, surgical complications, or the outcomes of ART following sperm retrieval.

The SRR of 33.3% in our study is relatively lower than that in other retrospective studies in the literature. This could be attributed to the small sample size of our study and the fact that all of our patients were azoospermic, whereas other studies included oligospermic individuals or patients for whom a pre-operative semen analysis was not achievable. Among the three patients in our series who had a positive sperm retrieval, two of them underwent ICSI resulting in two pregnancies and the birth of a healthy child. The utilization rate of preserved spermatozoa is generally low, as observed in various studies on male fertility preservation. Griasole et al. [68] found that only 24% of patients with TC used their preserved sperm, and among them, only 10% utilized the stored sperm for ART. In another prospective study involving 186 patients, the reasons for the low utilization of stored sperm were reported as spontaneous pregnancy (55%), the recovery of acceptable seminal fluid (28%), and patient mortality (18%) [69]. A more recent retrospective study of 59 patients who had their ejaculated sperm stored before cancer treatment reported an estimated utilization rate of stored sperm vials at 11.9%, with a spontaneous pregnancy rate of 22% [70].

Our study has certain limitations such as its retrospective and monocentric nature, resulting in the inclusion of a small sample size that did not allow us to perform a significant analysis, such as one on the presence of predictive factors for SR.

## 5. Conclusions

Onco-TESE proved to be a safe and effective option for improving fertility preservation in azoospermic males affected by TC while ensuring that oncological treatment remained uncompromised. It is imperative to explore testicular function through a semen analysis and to embrace cryopreservation as standard practice in the clinical setting prior to orchiectomy. To date, no reliable predictive factors exist to determine the success of sperm retrieval through onco-TESE. However, further studies are warranted to expand our knowledge in this field, as, to date, no high-level evidence is available.

## Figures and Tables

**Table 1:** Descriptive characteristics for the cohort of 9 patients who underwent onco-mTESE.

Variables	Total
Number of patients, *n* (%)	9
Median age, years (IQR)	34 (29–36)
Smoking habit, *n* (%)	4 (44.4)
Diabetes, *n* (%)	0 (0-0)
BMI, *n* (IQR)	23 (22–25)
Infertility Risk Factors	
Cryptorchidism, *n* (%)	1 (11.1)
Microlithiasis, *n* (%)	2 (22.2)
Inguinal hernioplasty, *n* (%)	2 (22.2)
Previous TC ^1^, *n* (%)	3 (33.3)
Azoospermia, *n* (%)	9 (100)
LH, IU/mL (IQR)	6.0 (2.5–10.3)
FSH, IU/L (IQR)	18.3 (11.7–41)
Testosterone, nmol/L (IQR)	18 (15.5–21.0)
Follow-up, months (IQR)	50 (30–113)

^1^ Testicular cancer.

**Table 2:** Patients’ pre-operative characteristics and post-operative histopathology reports.

							Sperm Retrieval				
Patient	Infertility Risk Factors	FSH IU/L	LH IU/L	Testosterone pg/mL	Tumor Volume, mL	Testis Volume, mL	Testis with TC ^1^	Healthy Testis	Histology	Cancer Type	Stage
1	-	8.8	6	4.26	5	13	yes	yes	normal spermatogenesis	SGCT ^2^	Stage IA
2	Inguinal hernioplasty	13.8	7	6.5	4	7	no	no	maturation arrest	NSGCT ^3^	Stage IB
3	TC history	45.3	13.6	9	5	8	no	N/A	SCOS ^4^	NSGCT	Stage IB
4	Cryptorchidism Microlithiasis TC history	22.5	0.1	4.21	6	8	yes	N/A	severe hypo ^5^	SGCT	Stage IA
5	TC history	40	3	6.5	3	8	no	N/A	SCOS	NSGCT	Stage IIB
6	Microlithiasis	42	2	7	5	14	no	no	SCOS	SGCT	Stage IIA
7	-	9.6	6	4.26	6	13	no	no	severe hypo	SGCT	Stage IIB
8	Inguinal hernioplasty	13.8	7	6.5	5	7	no	no	maturation arrest	NSGCT	Stage IA
9	-	45.3	13.6	9	4	10	no	yes	severe hypo	NSGCT	Stage IIA

^1^ Testicular cancer; ^2^ seminomatous germ cell tumor; ^3^ non-seminomatous germ cell tumor; ^4^ Sertoli-only-cell syndrome; ^5^ hypospermatogenesis.

**Table 3:** Pathological and histological features of the 9 onco-mTESE procedures performed.

Variables	Total
Number of patients, *n* (%)	9
Testis volume, mL (IQR)	8 (7.5–13)
Tumor volume, mL(IQR)	5 (4–5.5)
Positive Tumor Markers	
AFP, *n* (%)	0 (0)
Β-HCG, *n* (%)	3 (33.3%)
LDH, *n* (%)	2 (22.2%)
Cancer Type	
SGCT ^1^, *n* (%)	4 (44.4)
NSGCT ^2^, *n* (%)	5 (56.6)
GCNIS ^3^ presence *n* (%)	7 (77.8)
Sperm retrieval, *n* (%)	3 (33.3)
Vials stored, *n* (IQR)	5 (2–8)
Histology	
Normal spermatogenesis, *n* (%)	1 (11.1)
Severe hypospermatogenesis, *n* (%)	3 (33.3)
SCOS ^4^, *n* (%)	3 (33.3)
MA ^5^, *n* (%)	2 (22.2)
Johnsen score, *n* (IQR)	2 (1.5–7)
Post-operative complications	0 (0)

^1^ SGCT = seminomatous germ cell tumor; ^2^ NSGCT = non-seminomatous germ cell tumor; ^3^ germ cell neoplasia in situ; ^4^ Sertoli-only-cell syndrome; ^5^ maturation arrest.

**Table 4:** Studies reporting results on onco-TESE for TC.

Study	Study Type	Sample Size	AZO ^1^ *n*	OAT ^2^ or Crypt ^3^ *n*	Others ^4^ *n*	Cancer Type	Surgery	SRR ^5^ *n* (%)	ART ^6^
Res et al., 2000 [46]	Case report	1	1	-	-	SGCT ^7^	TESE ^12^	1 (100)	1 LB ^14^
Schrader et al., 2003 [47]	Retrospective study	31	31	-	-	8 SGCT, 6 NSGCT ^8^, 7 HD ^9^, 10 NHL ^10^	TESE	14 (45.2)	1 miscarriage and 1 LB
Binsaleh et al., 2004 [49]	Case report	2	2	-	-	2 NGCT	Mtese ^13^	1 (50)	1 pregnancy
Carmignani et al., 2007 [50]	Case report	4	4	-	-	2 SGCT; 2 NSGCT	mTESE	3 (75)	1 ICSI ^15^
Descombe et al., 2008 [51]	Case report	2	1	1	-	1 SGCT; 2 NSGCT	TESE	2 (100%)	1 LB
Hallak et al., 2009 [52]	Retrospective study	5	5	-	-	1 SGCT, 5 NGTT ^11^	mTESE	4 (80.0)	1 LB
Safsaf et al., 2011 [53]	Case report	1	1	-	-	1 SGCT	TESE	1 (100)	None
Furuhashi et al., 2013 [54]	Retrospective study	6	4	2	-	3 SGCT, 2 NSGCT, 1 NGTT	TESE	4 (66.7)	1 pregnancy
Berookhim and Mulhall 2014 [55]	Retrospective study	21	11	2	8	N/A	TESE	8 (38.1)	None
Haddad et al., 2014 [56]	Case report	1	-	1	-	1 SGCT	TESE	1 (100)	None
Roque et al., 2015 [57]	Case report	1	1	-	-	1 SGCT	TESE	1 (100)	1 LB
Luján et al. 2016 [58]	Case report	1	1	-	-	1 SGCT	TESE	1 (100)	1 LB
Pindoria et al., 2016 [59]	Case report	1	1	-	-	1 epididymal papillary cystadenocarcinoma	TESE and mTESE	1 (100)	1 LB
Tsutsumi et al., 2017 [60]	Case report	2	2	-	-	2 SGCT	TESE	1 (50)	None
Kuroda et al., 2018 [61]	Case report	1	1	-	-	1 SGCT	mTESE	1 (100)	None
Sener et al., 2018 [62]	Case report	1	-	1	-	1 SGCT	TESE	1 (100)	None
Takeshima et al., 2019 [63]	Case report	1	1	-	-	1 epidermal cyst	TESE	1 (100)	None
Hayashi et al., 2020 [64]	Case report	1	1	-	-	1 SGCT	TESE	1 (100)	1 LB
Blecher et al., 2021 [65]	Retrospective study	13	12	1	-	3 SGCT; 4 NSGCT; 4 NGTT; 2 benign lesions	mTESE	10 (83)	1 miscarriage; 6 LB
Giwerc et al., 2021 [66]	Retrospective study	24	9	5	10	13 SGCT; 11 NSGCT	TESE	14 (58.3)	1 ICSI
Scott et al., 2021 [48]	Retrospective study	9	9	-	-	N/A	mTESE	7 (78%)	None
Symeonidis et al., 2021 [67]	Case report	1	1	-	-	NSGCT	mTESE	1 (100)	None
Cirigliano et al. (current study)	Retrospective study	9	9	-	-	4 SGCT; 5 NSGCT	mTESE	3 (33.3)	1 miscarriage 1 LB

^1^ Azoospermia; ^2^ oligoasthenoteratospermia; ^3^ cryptozoospermia; ^4^ sperm collection failures, sperm collection refusal, anejaculation; ^5^ sperm retrieval rate; ^6^ Assisted Reproductive Technology; ^7^ seminomatous germ cell tumor; ^8^ non-SGCT; ^9^ Hodgkin’s disease; ^10^ non-Hodgkin’s lymphoma; ^11^ nongerminal testicular tumor; ^12^ testicular sperm extraction; ^13^ microsurgical TESE; ^14^ live birth; ^15^ intracytoplasmic sperm injection.

## Data Availability

Additional data are available from the corresponding author on reasonable request.
